# Substance use and associated factors among preparatory school students in Bale Zone, Oromia Regional State, Southeast Ethiopia

**DOI:** 10.1186/1477-7517-11-21

**Published:** 2014-08-09

**Authors:** Nagasa Dida, Yibeltal Kassa, Teshome Sirak, Ephrem Zerga, Tariku Dessalegn

**Affiliations:** 1 Department of Public Health, College of Medicine and Health Sciences, Madawalabu University, Bale Robe, Ethiopia; 2 Department of Nursing, College of Medicine and Health Sciences, Madawalabu University, Bale Robe, Ethiopia; 3 Department of Psychology, School of Behavioral Sciences, Madawalabu University, Bale Robe, Ethiopia; 4 Department of Sociology, School of Behavioral Sciences, Madawalabu University, Bale Robe, Ethiopia

**Keywords:** Substance use (khat, alcohol, cigarette, and shisha), Associated factors

## Abstract

**Introduction:**

The use of cigarettes, alcohol, khat, and other substances is a worldwide threat which especially affects young people and which is also common among the youth of Ethiopia. However, its prevalence and associated factors have not been addressed well yet. Thus, this study aimed to assess the prevalence and associated factors of substance use among preparatory school students in Bale Zone, Oromia Regional State, Southeast Ethiopia.

**Methods:**

An institutional-based cross-sectional study was conducted among 603 randomly selected students from five of eight preparatory schools of Bale Zone, Oromia Regional State, Southeast Ethiopia, in March 2013. The sample size was calculated by a single population proportion formula and allocated proportionally for the schools based on the number of students. A pretested structured questionnaire was used to collect the data. The data were analyzed using SPSS version 16.0. Descriptive, bivariate, and multivariate logistic regressions were employed to identify the predictors of substance use.

**Result:**

The overall current prevalence of substance use among the respondents was 34.8% (210). Specifically, 23.6% (102) and 4.6% (28) of the respondents chewed khat and smoked cigarette, respectively. Sex, age, and substance use status of the respondents’ father, mother, siblings, and best friend had an association with substance use. Male respondents were about ten times more at risk of practicing substance use compared to female respondents [adjusted odds ratio (AOR) 11.37, 95% confidence interval (CI) 4.42–29.23]. Respondents whose sibling(s) smokes cigarette were four times more likely to use substance (AOR 4.44, 95% CI 1.11–17.79). Respondents whose best friend chews khat were 11 times more likely to use substance when compared with those whose best friend does not practice the given factor (AOR 11.15, 95% CI 4.43–28.07).

**Conclusion:**

Respondents whose family uses one or more substances were more likely use substance(s). Respondents whose best friend uses substance(s) were more prone to practice substance use. Fifteen years of age of the respondents was the critical age when they began to practice substance use. Sex and family of the respondents were the predicting factors for them to practice substance use or not. Hence, health extension workers and district health workers should tackle substance use of the respondents through focusing the identified factors.

## Introduction

Substance use is a phenomenon which can be observed in different forms and displays all around the world [1]. The World Health Organization (WHO) estimates that there are about 2 billion people worldwide who consume alcoholic beverages and 76.3 million of them are affected with alcohol-related disorders. From a public health perspective, the global burden related to alcohol consumption, both in terms of morbidity and mortality, is considerable in most parts of the world [2]. There is a growing epidemic of tobacco use among adolescents in the developing world [3]. Khat (*Catha edulis*) has been used in African countries for centuries as a mild stimulant. For most youths, chewing khat is considered as a method of increasing energy and elevating mood in order to improve work performance [4].

Harmful effects can occur with any psychoactive substance use. Early initiation of substance use is found to be associated with an increased risk of developing addiction and adulthood dependence. The WHO report on substance use in South Africa showed that negative consequences had been brought about on the society. Criminal activity, a neglect of social responsibilities, disease, injury, and death are results of this substance use. In general, it has been shown that the use of cigarettes, alcohol, and other substances is a worldwide threat which affects young people [5].

In some parts of Ethiopia like Bale and Harar, khat chewing in communities in which smoking is common has been seen as a social custom that dates back thousands of years. Many students also considered khat chewing as a method of improving their reading ability. However, no activity has been seen on the reduction of substance use [6,7].

The prevalence of substance use is different from country to country and time to time. Information on substance use and its associated factors have a significant input for researchers and policy makers to design an intervention strategy for health problems because of substance use [8,9]. However, this issue has not been addressed yet among school youth pupils of the study area. Thus, this study assessed the prevalence and associated factors of substance use among preparatory school students in Bale Zone, Oromia Regional State, Southeast Ethiopia.

## Methods

### Study setting and participation

An institutional-based cross-sectional study was conducted among 603 randomly selected preparatory school students of Bale Zone, in March 2013. Bale Zone is found in Oromia Regional State at 430 km away from Addis Ababa to Southeast Ethiopia. It is the largest zone of Oromia Regional State with an area of 62,555 km^2^. The zone is composed of 14.92% highland, 21.53% midland, and 63.55% lowland with an altitude of 300–4,377 km and annual rainfall of 900–1,400 mm. Of the total of eight preparatory schools in Bale Zone, five were randomly selected for the study. These selected preparatory schools were Batu Terara, Robe, Goro, Gololcha, and Ginnir.

The source population was students studying in all preparatory schools in Bale Zone, and the study population was students studying in preparatory schools during the period of data collection and sampled for interview. The sample size was allocated proportionally for five of the schools which were identified through simple random sampling. The sample size was determined by a single population proportion formula with the following assumptions: level of confidence of the study 95%, sampling error tolerated 5%, proportion (*P*) of Harar town high school students who chew khat 24.2% [9], nonresponse rate 10%, and design effect of 2.

### Instruments and data collection methods

A structured questionnaire covering substance use and its associated factors was adapted from different literatures that were pertinent to the topic [1,7,10]. The questionnaire has a total of 114 multiple choice questions. The domain of the tool addresses the prevalence of substance use (cigarette, khat, alcohol, and shisha) and its associated factors. The questionnaire was translated to the local language Afan Oromo and Amharic language by a language expert and retranslated by other persons to check its consistency. Training in the data collection process was given to data collection facilitators for 2 days. The tool was pretested on 5% of the sample size that were not from the schools selected for the study. The data were collected through a self-administered questionnaire.

### Data processing and analysis

The data were coded, entered, cleaned for completeness and inconsistencies, and analyzed using SPSS version 16.0 for Windows. Descriptive and bivariate logistic regressions were used to assess associated factors with the study variable. Regardless of the number of substances used by the respondents, they were indexed as users and nonusers for logistic regression analysis. Multivariate logistic regression was employed to identify predictors of substance use after adjusting for variables that were significant under bivariate logistic analysis; a corresponding *p* value of <0.05 was considered to be statistically significant.

### Ethical consideration

The ethical issue was approved by Madawalabu University Ethical Review Committee. A supportive letter was obtained from the University Research Directorate to all schools. Explaining the purpose of the study, verbal consent was obtained from the participants. The respondents had the right to fill out or refuse the questionnaire at all or partially. All the information given by the respondents has been used for research purposes only, and confidentiality was maintained by omitting the names of the respondents.

## Result

### Socio-demographic characteristics

A total of 603 preparatory school students participated in the study with a response rate of 97.9%. The mean age of the respondents was 18.4 (standard deviation (SD) ±1.55). The majority (94.9%, 572) of the respondents were living in the town. Sixty-two percent (372) of the respondents were Orthodox Christian by religion. Fifty-five percent (333) of the respondents were from grade 11, and 62% (373) of the respondents were males (Table 1).

**Table 1:** Socio-demographic characteristics of the respondents, Bale Zone, Oromia Regional State, Southeast Ethiopia, March 2013

**Variables**		**Frequency**	
		**Number (603)**	**Percentage (%)**
Place of birth	Town	314	52.1
	Rural	284	47.1
Current residence	Town	572	94.9
	Rural	27	4.5
Sex	Male	373	61.9
	Female	228	37.8
Age	15–19	440	85.6
	20–24	67	13.0
	25–29	7	1.4
Grade	Grade 11	333	55.2
	Grade 12	263	43.6
Religion	Orthodox	372	61.7
	Muslim	166	27.5
	Protestant	51	8.5
	Wakefata	9	1.5
	Others	3	0.5

### Distribution of substance use of the respondents

The overall current prevalence of substance use among the respondents was 34.8% (210). Specifically, 23.6% (102), 17.1% (142), 5.6% (34), and 4.6% (28) of the respondents drank alcohol, chewed khat, smoked shisha, and smoked cigarette, respectively, at the time of data collection, but 24.4% (147) of the respondents used only one of the substances (Table 2). The mean age at which the respondents had started substance use was 15.1 (SD ±3.6). Of the total substance users, 53.3% (112) were from grade 12 and 85.7% (180) were males (Table 3).

**Table 2:** Substance use distribution of the respondents, Bale Zone, Oromia Regional State, Southeast Ethiopia, March 2013

**Variables**		**Number**	**Percentage (%)**
Khat	Yes	102	17.1
	No	494	82.9
Alcohol	Yes	142	23.6
	No	460	76.4
Cigarette	Yes	28	4.6
	No	575	95.4
Shisha	Yes	34	5.6
	No	569	94.4
Number of substances used	1	147	24.4
	2	39	6.5
	3	15	2.5
	4	9	1.5

**Table 3:** Associated factors and predictors of substance use among preparatory school students

**Factors**		**Substance use**		**COR [95% CI]**	**AOR [95% CI]**
		**Yes**	**No**		
Sex	Male	180	193	6.16 (3.00–9.50)*	11.37 (4.42–29.23)*
	Female	30	198	1.00	1.00
Age	15–19	151	289	1.00	1.00
	20–24	33	34	0.54 (0.32–0.90)*	0.90 (0.36–2.22)
	25–29	3	4	0.70 (0.15–3.15)	0.38 (0.03–5.48)
Current residence	Town	201	371	1.29 (0.55–2.99)	
	Rural	8	19	1.00	
Religion	Muslim	60	106	1.00	
	Orthodox	136	236	0.98 (0.67–1.44)	
	Protestant	8	43	3.04 (1.34–6.90)	
	Wakefata	4	5	0.71 (0.18–2.74)	
	Others	2	1	0.28 (0.03–3.19)	
Grade	11	97	237	1.80 (1.28–2.52)*	
	12	113	154	1.00	
Father smokes cigarette	Yes	38	35	2.24 (1.36–3.67)*	0.92 (0.33–2.58)
	No	172	355	1.00	1.00
Father drinks alcohol	Yes	98	94	2.80 (1.95–4.00)*	4.21 (2.06–8.62)*
	No	111	298	1.00	1.00
Mother drinks alcohol	Yes	21	7	6.13 (2.56–14.67)*	5.40 (0.89–32.89)
	No	188	384	1.00	1.00
Father chews khat	Yes	55	34	3.73 (2.34–5.95)*	4.14 (1.71–10.02)*
	No	154	355	1.00	1.00
Mother chews khat	Yes	19	7	5.50 (2.27–13.31)*	0.18 (0.03–1.24)
	No	190	385	1.00	1.00
Sibling(s) smoke cigarette	Yes	20	6	7.36 (2.88–18.82)*	4.44 (1.11–17.79)*
	No	115	254	1.00	1.00
Sibling(s) chew khat	Yes	55	34	4.56 (2.80–7.41)*	1.24 (0.46–3.34)
	No	98	276	1.00	1.00
Sibling shisha use status	Yes	10	5	4.33 (1.46–12.85)*	0.90 (0.15–5.42)
	No	171	370	1.00	1.00
Sibling alcohol use status	Yes	49	29	4.25 (2.56–7.05)*	1.58 (0.58–4.34)
	No	115	289	1.00	1.00
Best friend smokes cigarette	Yes	27	15	3.71 (1.93–7.14)*	0.12 (0.03–0.54)*
	No	183	377	1.00	1.00
Best friend drinks alcohol	Yes	45	22	4.57 (2.66–7.87)*	1.17 (0.31–4.44)
	No	165	369	1.00	1.00
Best friend drunk at least once a week	Yes	46	21	4.96 (2.87–8.57)*	3.90 (1.34–11.36)*
	No	164	371	1.00	1.00
Best friend uses shisha	Yes	24	10	4.92 (2.30–10.49)*	1.06 (0.18–6.42)
	No	186	381	1.00	1.00
Best friend chews khat	Yes	77	28	7.55 (4.69–12.15)*	11.15 (4.43–28.07)*
	No	133	365	1.00	1.00

*p value <0.05.

### Factors associated with substance use of the respondents

To identify associated factors of substance use among preparatory school students, bivariate logistic regression was used, and multivariate logistic regression was applied to identify the predictors of substance use at a *p* value less than 0.05.

Accordingly, sex, age, and substance use status of the respondents’ father, mother, and siblings had an association with substance use. Male respondents were six times more likely to use substances compared to female respondents. Respondents whose father smokes cigarette were 2.24 times more likely to use substance(s) compared to respondents whose father seldom uses any of the substances. Similarly, respondents whose mother chews khat and sibling(s) smokes cigarette were five times (crude odds ratio (COR) 5.50, 95% confidence interval (CI) 2.27–13.31) and seven times (COR 7.36, 95% CI 2.88–18.82) more likely to use substances, respectively, when compared with those whose mother and sibling(s) seldom use these substances (Table 3).

When factors associated with substance use were adjusted for confounding factors: sex, respondents’ father who drinks alcohol and chews khat, and respondents’ sibling(s) who smokes cigarette showed a significant association with substance use of the respondents. Similarly, substance use of respondents’ best friend also had a significant association with substance use of the respondents. Respondents whose father drinks alcohol were four times (adjusted odds ratio (AOR) 4.21, 95% CI 2.06–8.62) more likely to use at least one of the substances. Additionally, respondents whose best friend (the friend who approaches the respondents more) drinks alcohol at least once a week were about four times (AOR 3.90, 95% CI 1.34–11.36) more likely to use substances when compared with those whose best friend does not drink (Table 3).

## Discussion

This study assessed the prevalence and associated factors of substance use among preparatory school students of Bale Zone, Oromia Regional State, Southeast Ethiopia. The overall current prevalence of substance use among the respondents was 34.8% (210). Specifically, 24% (102) and 17.1% (142) of the respondents drank alcohol and chewed khat, respectively.

A study done among students at northwest of Ethiopia stated that the lifetime prevalence rate of cigarette smoking and khat chewing was 13.1% and 26.7%, respectively. The current prevalence of cigarette smoking was found to be 8.1% and that of khat chewing 17.5%, which was higher than and nearly similar to the prevalence rate of this study, respectively [7]. In Saudi Arabia, the overall prevalence of khat chewing among students was similar to this study [11]. By the year 2006, the prevalence of smoking among Jamaican school-going adolescents was 16.7%, which was higher by more than 10% when compared with the prevalence of cigarette smoking of this study [12]. Similarly, a study conducted among Indian higher secondary school students showed a higher prevalence of substance use of 551 (54%, 95% CI 42%–67%) which might be due to the scope of the study which covered more substances in the case of India [13]. In Nigeria high school students, the overall lifetime prevalence of substance use (caffeine, analgesics, and antimalarial) was higher (87.3%) which could be because of the vast scope of the study [14]. Harare, Zimbabwe, high school students also showed a higher prevalence of cigarette smoking of 28.8% (95% CI 25.3%–32.3%) [3].

A study conducted in Dire Dawa, Ethiopia, high school students showed a higher lifetime prevalence of alcohol drinking (34.2%), cigarette smoking (13%), and shisha smoking (12.8%) but a similar prevalence of khat chewing (18.4%) when compared with this study [10]. The discrepancy might be because the study in Dire Dawa covered grades 9 to 12, but the current study included preparatory schools only. The lifetime prevalence of khat chewing of Harar, Ethiopia, high school students was 24.2%, which was similar to this study [15].

The mean age at which Bale Zone preparatory school students started substance use was 15.1 (SD ±3.6) years. The median age at first substance use of Indian higher secondary school students was 15.5 years, which was similar to this study [13]. Zimbabwean higher school students also started smoking earlier [3]. It was also similar to the first khat chewing mean (SD) age of Harar, Ethiopia, high school students which was 15.1 (2.33) years [15].

Through bivariate analysis, gender, age, and substance use status of the respondents’ father, mother, siblings, and best friend showed a statistically significant association with substance use of the respondents. Marital status and intimacy relationship of the respondents’ family (father and mother) did not associate with substance use of the respondents. But among Brazilian school adolescents, marital status and intimacy relationship of their family had a significant association with substance use. Adolescents whose parents were divorced reported more than 50% greater in using substance than those whose parents lived together (prevalence ratio (PR) 1.55, 95% CI 1.26–1.90) [16,17].

In Brazilian school adolescents, the presence of household members who drink excessively was statistically significant for drug use of the subject (PR 1.50, 95% CI 1.19–1.90) and the presence of other drug users in the household was also statistically significant (PR 1.98, 95% CI 1.42–2.76) which was also significantly associated in this study [17]. Respondents whose father smokes cigarette, whose mother chews khat, and whose sibling(s) smokes cigarette were 2.24, 5.5, and 7.36 times more likely to use substances, respectively, when compared with those whose father, mother, and sibling(s) seldom use these substances, nearly similar to Brazilian school adolescents who were 1.5 times more likely to use substance in the presence of household members who drink alcohol [17]. Among Jamaican school-going adolescents, having smoker parents made them to smoke cigarette 2.38 times more compared with students whose parents were nonsmokers (OR 2.38, 95% CI 1.80–3.16) [12].

Male respondents of this study were six times more likely to use substances which was less among Jamaican school-going adolescents who smoke (OR 2.04, 95% CI 1.56–2.68) [17]. Indian high school students who used substance ever were more likely to be males (3.0, 95% CI 2.4–39) [13]. But the prevalence of cigarette smoking was higher among Harare high school male students which was estimated to be 18.5% (95% CI 14.3%–23.3%) compared to female students [3].

A study conducted in Harar, Ethiopia, high school students showed that male students had two times higher odds of chewing khat compared to female students (OR 2.10, 95% CI 1.50–2.93) which was lower by 4 compared to this study [15].

Age category which was statistically significant in the case of Harar, Ethiopia, high school (OR 1.31, 95% CI 1.16–1.49) but not associated in this study (OR 0.90, 95% CI 0.36–2.22) which might be from the inclusion of grade 9 and 10 students [15].

After adjusting for variables associated under bivariate analysis, sex, respondents’ father who drinks alcohol and chews khat, and respondents’ sibling(s) who smokes cigarette showed a significant association with substance use of the respondents. Similarly, substance use of the respondents’ best friend also showed a significant association with substance use.

Respondents whose father drinks alcohol and chews khat and whose sibling(s) smokes cigarette were four times more likely to use substances (OR 4.21, 95% CI 2.06–8.62; OR 4.14, 95% CI 1.71–10.02; and OR 4.44, 95% CI 1.11–17.79, respectively) compared with respondents whose father seldom uses any of the substances. Also, among Indian high school students, substance use of family members showed a positive contribution to substance use of the students. Students whose father and sibling(s) use substances were two times more likely to use substance (OR 2.0, 95% CI 1.6–2.7 and OR 2.1, 95% CI 1.5–3.0, respectively) than respondents whose father and sibling(s) do not use substance [13].

Having a best friend who drinks alcohol at least once a week made the respondents to practice substance use four times more (OR 3.9, CI 1.34–11.36) when compared with those whose best friend does not use any of the substances. Similarly, having a friend who drinks alcohol made Zimbabwean high school students smoke about six times more (OR 5.7, 95% CI 2.9–11.5) [3]. Harar, Ethiopia, high school students who have friends who chew khat were eight times more likely to practice khat chewing when compared to those who do not have (OR 7.93, 95% CI 5.40–11.64) which was higher when compared with this study which might be due to socio-demographic differences of the study area [13].

As the data collection method was self-administered for self-report, it might have contributed to underreporting or overreporting. The time of the data collection period, which was when the students prepared for an entrance exam, could have made the students rush over the questionnaire which might affect the result of the study.

## Conclusion

The current substance use prevalence of preparatory school students of Bale Zone is high. Fifteen years of age of the respondents is the critical age at which they start to use substances. The students’ family was their model for practicing substance(s) or not.

Gender, respondents’ family (father and/or sibling(s)) who uses substance(s), and respondents’ best friend were the predictors of the substance use of the students. Hence, health extension workers and district health workers should tackle substance use of the respondents through focusing the identified factors.

## Competing interests

The authors declare that they have no competing interests.

## Authors’ contributions

ND and YK conceived and designed the study. TS was involved in the conception. ND and YK analyzed the data. ND prepared the manuscript. YK critically reviewed the manuscript. EZ and TD assisted in the data collection and reviewed the manuscript. All authors have read and approved the final manuscript.
